# Reconstruction of residents’ thyroid equivalent doses from internal radionuclides after the Fukushima Daiichi nuclear power station accident

**DOI:** 10.1038/s41598-020-60453-0

**Published:** 2020-02-27

**Authors:** Takashi Ohba, Tetsuo Ishikawa, Haruyasu Nagai, Shinji Tokonami, Arifumi Hasegawa, Gen Suzuki

**Affiliations:** 1 Department of Radiation Health Management, School of Medicine, Fukushima Medical University, Fukushima-city, Fukushima 9601295 Japan; 2 Radiation Medical Science Centre for the Fukushima Health Management Survey, Fukushima Medical University, Fukushima-city, Fukushima 9601295 Japan; 3 Department of Radiation Physics and Chemistry, School of Medicine, Fukushima Medical University, Fukushima-city, Fukushima 9601295 Japan; 4 Environment and Radiation Science Division, Japan Atomic Energy Agency, Tokai-village, Ibaraki, 3191195 Japan; 5 Department of Radiation Physics, Institute of Radiation Emergency Medicine, Hirosaki University, Hirosaki-city, Aomori 0368564 Japan; 6 Department of Radiation Disaster Medicine, School of Medicine, Fukushima Medical University, Fukushima-city, Fukushima 9601295 Japan; 7 International University of Health and Welfare Clinic, Ohtawara-city, Tochigi 3248501 Japan

**Keywords:** Environmental impact, Risk factors

## Abstract

There is concern among residents that their children might suffer from thyroid cancer in the near future after the Fukushima Daiichi nuclear power station (FDNPS) accident. However, the demographic and geographical distribution of thyroid equivalent doses was not thoroughly evaluated, and direct thyroid measurements were conducted only for 1,200 children, whose individual thyroid doses were assessed on the basis of those measurements accounting for the dynamics of radioiodine intake. We conducted hierarchical clustering analyses of 100 or 300 randomly sampled behavioural questionnaire sheets of children from each of seven municipalities in the evacuation area to reconstruct evacuation scenarios associated with high or low exposures to plumes. In total 896 behaviour records in the Fukushima Health Management Survey were analysed to estimate thyroid equivalent doses via inhalation, using a spatiotemporal radionuclides concentration database constructed by atmospheric dispersion simulations. After a decontamination factor for sheltering and a modifying factor for the dose coefficient—to reflect lower iodine uptake rate in Japanese—were applied, estimated thyroid equivalent doses were close to those estimated from direct thyroid measurement. The median and 95^th^ percentile of thyroid equivalent doses of 1-year-old children ranged from 0.6 to 16 mSv and from 7.5 to 30 mSv, respectively. These results are useful for future epidemiological studies of thyroid cancer in Fukushima.

## Introduction

The great east Japan earthquake and subsequent tsunami on 11 March, 2011 destroyed all electrical supply systems essential for cooling nuclear fuels in the Unit 1–3 rectors of the Fukushima Dai-ichi Nuclear Power Station (FDNPS) and caused a meltdown of fuel rods in the reactors^1^. The United Nations Scientific Committee on the Effects of Atomic Radiation (UNSCEAR) reported that 120 PBq of ^131^I, 29 PBq of ^132^Te/^132^I, and 9.6 PBq of ^133^I were released from the FDNPS^2^. Because radio-iodine tends to accumulate in—and irradiate—the thyroid gland, there is concern among residents that their children might suffer from thyroid cancer in the future. Therefore, the Fukushima Prefectural Government and Fukushima Medical University (FMU) began an ultrasound thyroid examination campaign in October 2011 for about 360,000 residents who were less than 19 years old at the time of the accident^3^. As of March 2019, 218 suspected or definite thyroid cancers had been found^4^. There is ongoing debate in Japan as to whether these observed thyroid cancers are radiation-induced or not^5–9^.

Knowledge of thyroid equivalent dose (TED) is essential for evaluating a causal association of thyroid cancer with the FDNPS accident. After the accident, thyroid measurements were conducted with a NaI (Tl) scintillation survey meter, NaI (Tl) spectrometer, or whole body counter (WBC) equipped with germanium detectors^10,11^. However, only about 1,200 children received these thyroid measurements, too few to accurately estimate the distribution of TEDs among evacuees who lived around the FDNPS. Therefore, in the UNSCEAR 2013 Report, thyroid absorbed doses were simulated for residents of three age categories in each municipality by an atmospheric transport, diffusion, and deposition model (ATDM) using information on the release rates of radionuclides from the reactors and their physicochemical properties, *i.e*., source term^2^, and eighteen representative evacuation scenarios from Akahane *et al*.^12^ (Supplementary Table 1). Since only one or two representative evacuation scenarios per municipality were proposed by Akahane *et al*.^12^, the distribution of childhood thyroid absorbed doses in each municipality was imprecise. In the present study, we conducted hierarchical clustering analyses of 100 or 300 randomly sampled behavioural questionnaire sheets of children from each of seven municipalities in the evacuation area to represent evacuation scenarios associated with high and low exposures to radioactive plumes. At the same time, childhood TEDs were estimated based on the questionnaire sheets under the assumption that all age groups evacuated following 100 or 300 evacuation scenarios in each municipality. The spatiotemporal distribution of ^131^I concentrations in air was simulated by the World-wide version of System for Prediction of Environmental Emergency Dose Information (WSPEEDI), a kind of ATDM simulation, developed by the Japanese Atomic Energy Agency (JAEA)^13^. This dataset was constructed after the refinement of the source term and ATDM simulations with many efforts such as the improvement of ATDM, application of new analysis method, utilization of new monitoring data, and it reproduced both the air concentrations at monitoring points and surface depositions by airborne monitoring much better than the previous studies including ATDM simulations used in the UNSCEAR 2013 Report. As plumes released from the FDNPS were rich in short-lived radionuclides, especially in the early stage of the accident^14,15^, TEDs via inhalation of ^132^Te/^132^I and ^133^I were also estimated in the present study.

## Methods

### Data settings and ethical issues

As reported elsewhere, FMU has been conducting the Basic Survey of Fukushima Health Management Survey (FHMS) since 2011, in which information on residents’ whereabouts from 11 March to 11 July is collected by self-administered questionnaires^16^. In the present analyses, a detailed version of the questionnaire encompassing the period 11 to 25 March, 2011, was used^16^. As of 2014, a total of 541,653 residents answered the questionnaire out of 2,055,533 people who were in Fukushima prefecture at the time of accident^16^. After approval was obtained from the Institutional Review Boards of the International University of Health and Welfare (IUHW) (13-B-185, August 2016, 13-B-339, March 2019) and FMU (No. 29100, August 2018, No. 29100-003, July 2019), individual questionnaire data on residents less than 20 years of age at the time of the accident were randomly selected from the FHMS database and provided to us as an anonymized data set. We performed all methods in accordance with the national ethical guidelines for epidemiological studies and the relevant institutional regulations. According to the guidelines, getting new informed consent from a questionnaire provider is not required for the present study. Instead, an optout option should be announced for the providers via the WEB pages of FMU and IUHW. The data set comprised the following items: age, gender, location of residence (excluding details of the address, such as house number), places visited (including latitude and longitude), length of time spent indoors and outdoors, and travelling time. As shown in Table 1, 100 data sets each from Okuma, Naraha, Tomioka, Futaba, and Namie Towns, 100 data sets from Iitate Village, and 300 data sets from Minamisoma City were obtained. Because of 2 incomplete data sets, 98 data sets were analysed for Naraha Town. One data set from Minamisoma City and one from Futaba Town reported unrealistic movements possibly caused by a coding error, where children moved to very distant places immediately or within an unrealistically short time; these two data sets were omitted from the analyses. Thus, 896 data sets from 7 municipalities were analysed (Table 1) . As for Minamisoma City, 63 evacuees from Odaka Ward and 236 evacuees from Haramachi and Kashima Wards were analysed separately, as only Odaka Ward in Minamisoma City was under evacuation orders on 12 March, 2011 (Table 1).

**Table 1:** Number of evacuation-behaviour questionnaire sheets obtained from the Basic Survey of Fukushima Health Management Survey.

Municipality	Futaba Town	Tomioka Town	Naraha Town	Okuma Town	Namie Town	Minamisoma City	Iitate Village
Number of request	100	100	100	100	100	300	100
Actually received sheets	100	100	99	100	100	300	100
Excluded	1 (loss of evacuation route)	—	1 (evacuation records during 23–25 March)	—	—	1 (loss of evacuation route)	—
Number of analysis	99	100	98	100	100	299	100
				Classification	Odaka Ward	Haramachi/Kashima Wards	
				Number of analysis	63	236	

First, information on personal daily whereabouts was reorganized by one-hour segments from 11 March to 25 March, 2011, and places stayed or visited were identified by latitude and longitude. Next, the one-hour-interval data were integrated into 6-hour segments (6-HS), *i.e*., AM1 (0 a.m. to 6 a.m.), AM2 (6 a.m. to 0 p.m.), PM1 (0 p.m. to 6 p.m.), and PM2 (6 p.m. to 0 a.m.) for the sake of simplicity as well as for reducing the uncertainty inherent in hourly estimates of radionuclides by WSPEEDI, as noted below. Thus, individual dose was calculated as an integral of data from 56 6-HS from 12 March to 25 March, as radionuclides began leaking from 12 March and WSPEEDI simulated radionuclide concentrations were available from that day.

### Clustering analyses

Stepwise regression analysis revealed that about five 6-HS contributed to most of the total TEDs, as shown in Supplementary Fig. 1. Then, for 3–5 segments from each municipality hierarchical clustering analysis was performed by the Ward method using JMP^®^ 14.2.0 (SAS Institute Inc. NC, USA). If clustering based on the doses in the 6-HS was not satisfactory, a few key locations (landmarks) were included. Several representative evacuation scenarios for each municipality are shown in Supplementary dataset 1.

### Estimation of TED via inhalation

Average ^131^I concentrations (Bq/m^3^) of three chemical forms (methyl iodide, elemental vapor, and particulate iodine) at a height of 1 m in each 6-HS were calculated for 152 landmarks in Fukushima Prefecture (Supplementary Fig. 2) by referring to a spatiotemporal radionuclides concentration database constructed by WSPEEDI simulations (WSPEEDI_2019DB) using latitude and longitude as a key^13^. Many landmarks were selected for Minamisoma City, Iwaki City, and Namie Town because residents or evacuees in these areas suffered from radioactive plumes and radionuclide concentrations changed sharply from one 1-km-grid location to neighbouring grids in these areas. A Python (v3.6.7, Python Software Foundation. OR, USA) programme was kindly provided by Dr. Kurihara, QST-NIRS, Chiba, Japan, a program that can extract data from WSPEEDI_2019DB written according to a NetCDF protocol and make data usable as a Microsoft^®^ Office Excel worksheet (an Excel data file showing the temporal pattern of radionuclides in 152 landmarks is provided in Supplementary dataset 2). Average ^131^I concentration for each individual’s 56 6-HS was calculated by using values at the nearest landmark. TED via inhalation (*E*_*Thyroid (inhal)*_) was calculated by formula (1),

1 $${{\rm{E}}}_{{\rm{Thyroid}}({\rm{inhal}})}=\mathop{\sum }\limits_{{\rm{i}}}^{56}\frac{{\rm{V}}}{4}\,\times \,({{\rm{C}}}_{{\rm{i}}-{\rm{p}}}{\times {\rm{e}}}_{{\rm{inhal}}-{\rm{Thy}}-{\rm{p}}}{+{\rm{C}}}_{{\rm{i}}-{\rm{el}}}{\times {\rm{e}}}_{{\rm{inhal}}-{\rm{Thy}}-{\rm{el}}}{+{\rm{C}}}_{{\rm{i}}-{\rm{met}}}{\times {\rm{e}}}_{{\rm{inhal}}-{\rm{Thy}}-{\rm{met}}}){\times {\rm{FC}}\times {\rm{DF}}}_{{\rm{shelter}}},$$

where *V* is age-specific total daily ventilation volume, *Ci-p*, *Ci-el*, and *Ci-met* are the average concentrations (Bq/m^3^) of ^131^I-particulate, ^131^I-elemental vapor, and ^131^I-methylated forms of 6-HS, respectively, and *e*_*inhal/Thy-p*,_ *e*_*inhal/Thy-el*,_ *e*_*inhal/Thy-met*_, are age-dependent TED conversion factors from ICRP publication 71 (Supplementary Table 2)^17^. As reported^13^, WSPEEDI-2019DB has simulated the ^131^I concentrations of ^131^I-particulate, ^131^I-elementalal vapor, and ^131^I-methylated forms separately with the source term of ^131^I assuming 50%-particulate, 20%-elemental, and 30%-methyl forms. *FC* is a correction factor for the dose coefficient because iodine uptake rate is 18.5% (SD 6.0%), lower than the 30% in the ICRP thyroid model, while thyroid volume in Japanese does not differ from that of ICRP reference man^18^. *DF*_*shelter*_ is a decontamination factor to reflect sheltering. In the present study we set FC to 0.62 (=18.5/30) and *DF*_*shelter*_ to 0.5, and the combined uncertainty interval of these two factors is simulated as described below. It is noted that for *Ci-p*, *Ci-el*, and *Ci-met* we used the average concentrations in each 6-HS to reduce uncertainty in plume arrival time, because the WSPEEDI simulation predicted a plume arrival at a suspended particulate matter (SPM) monitoring station in Haramachi, Minamisoma City a few hours earlier than the actual arrival time in monitoring data on 12 March 2011^13^.

TED via inhalation (*E*_*Thyroid (inhalation) with short*_) was calculated by formula (2),

2 $${{\rm{E}}}_{{\rm{Thyroid}}({\rm{inhalation}}){\rm{with}}{\rm{short}}}{={\rm{E}}}_{{\rm{Thyroid}}({\rm{inhal}})}\times {\rm{SF}},$$

where *SF* is a correction factor for short-lived radionuclides. As for the radioactive plume on 12–13 March, 2011, short-lived radionuclides other than ^131^I also contributed to TEDs^14,15^. Based on the radionuclide composition ratio on clothing of evacuees^15^, the total ^131^I TED of 1-year-old children is multiplied by 1.59 to calculate TED from ^131^I, ^132^I, ^133^I, and ^132^Te. Likewise, for the radioactive plumes on 15–16 March, 2011, the total ^131^I TED of 1-year-old children is multiplied by 1.08.

### Estimation of TED via ingestion in Iitate village

Estimation methodology and doses estimated by taking into account contaminated tap water were reported elsewhere^19–21^. Because ingestion doses were much higher in Iitate village than in other evacuation municipalities, it is problematic to compare TEDs via inhalation in the present study with TEDs estimated based on direct thyroid measurements. Thus, for Iitate residents, TEDs from tap water were re-evaluated on the basis of 100 randomly sampled questionnaire sheets and combined with TEDs via inhalation. There are four water-supply sources in Iitate village: tap water supplied from (a) Takishita tap water processing plant (TWPP), (b) Hanatsuka TWPP, and (c) Tajiri TWPP, and (d) well water. Each TWPP supplied tap water of different ^131^I-contamination levels to dwellings in their own service area in Iitate village, and 30% of the dwellings in the area used well water^22^. TED via ingestion (*E*_*Thyroid-ingestion)*_) from 12–25 March 2011 was calculated by formula (3),

3 $${{\rm{E}}}_{{\rm{Thyroid}}({\rm{ingestion}})}=\mathop{\sum }\limits_{{\rm{j}}}^{14}{{\rm{V}}}_{{\rm{tap}}}\,{\times {\rm{C}}}_{{\rm{tap}}}\,{\times {\rm{e}}}_{{\rm{ing}}/{\rm{thy}}}\times {\rm{FC}}\times {\rm{Sf}}\times \frac{{{\rm{X}}}_{{\rm{j}}}}{3},$$

where tap water consumption volume (*V*_*tap*_) is set to 0.76 L/d, 1.03 L/d, and 1.65 L/d for 1-, 5-, and 10-year-old children, respectively, as reported elsewhere^20,21^; *C*_*tap,j*_ is ^131^I concentration (Bq/L) of tap water supplied from one of three TWPP; *e*_*ing/thy*_ is an age-specific TED conversion factor for ^131^I based on ICRP publication 67^23^; *FC* is the correction factor for TED described before; *Sf* is a correction factor for correcting tap water usage fraction; and *X*_*j*_ is number of meals taken in Iitate village on day *j*. If children evacuated after breakfast or lunch, ingestion dose on that day is considered to be 1/3 or 2/3 of total daily dose, respectively, and ingestion dose obtained in the destination area from the remaining daily meal(s) is added to daily dose.

### Uncertainty of correction factors

The ingestion and inhalation dose calculation formulas both contain two correction factors. The uncertainty interval for estimated dose by ingestion and inhalation combined was estimated by Monte Carlo simulation. As shown in Supplementary Table 3, we adopted results of *DF*_*shelter*_ reported by Hirouchi *et al*.^24^, and *DF*_*shelter*_ after the FDNPS accident in Fukushima prefecture is estimated by a triangular distribution from 0.1 to 0.95, with a peak at 0.5. The *DF*_*shelter*_ scores in houses vary with construction year as building codes changed in 1980 and 1992. Thus, the fractions of houses constructed under different building codes in 2011 in Fukushima prefecture are estimated using National Statistics for 2008 in Fukushima prefecture^25^, and construction-year-averaged *DF*_*shelter*_ is calculated (Supplementary Table 3). Other factors influencing *DF*_*shelter*_ are wind speed and elapsed time since sheltering. As for wind speed, a radioactive plume released by venting at 14:30 and by a hydrogen explosion at 15:36 on 12 March, 2011, reached an SPM monitoring station in Minamisoma city about 24 km from FDNPS at 20:00 or later on that day^26^. If the wind blew straight, the wind speed is estimated to have been 1.3–1.5 m/s. In this study, the central estimate of wind speed was set to 2.5 m/s, and elapsed time at 6 hours. Then construction-year averaged *DF*_*shelter*_ becomes 0.5 (Supplementary Table 3). In a Monte Carlo simulation to estimate the combined uncertainty of *FC* and *DF*_*shelter*_ in formula (1), we set the probability density distribution of *FC* to be normal with parameters (18.6+/−6.0)/30% and *DF*_*shelter*_ as a triangular distribution from 0.1 to 0.95 with a peak at 0.5. Monte Carlo simulation was repeated 100,000 times using a Latin Hypercube sampling method by the Crystal Ball software (release 11.1.2.3.500, Kozo Keikaku Engineering Inc., Tokyo, Japan). Likewise, the combined uncertainty of *FC* and *Sf* in formula (2) was simulated by setting *FC* as having a normal distribution with (18.6+/−6.0)/30% and *Sf* as having a binomial distribution with denominator 100 and expected proportion 0.3.

### Ethical approval and informed consent

The ethics committee of International University of Health and Welfare approved this study (13-B-185, 13-B-339). And the ethics committee of FMU approved this study (No. 29100, No. 29100-003).

## Results

### Representative evacuation patterns and ^131^I-TEDs via inhalation for youths less than 20 years old

**Figure 1:**
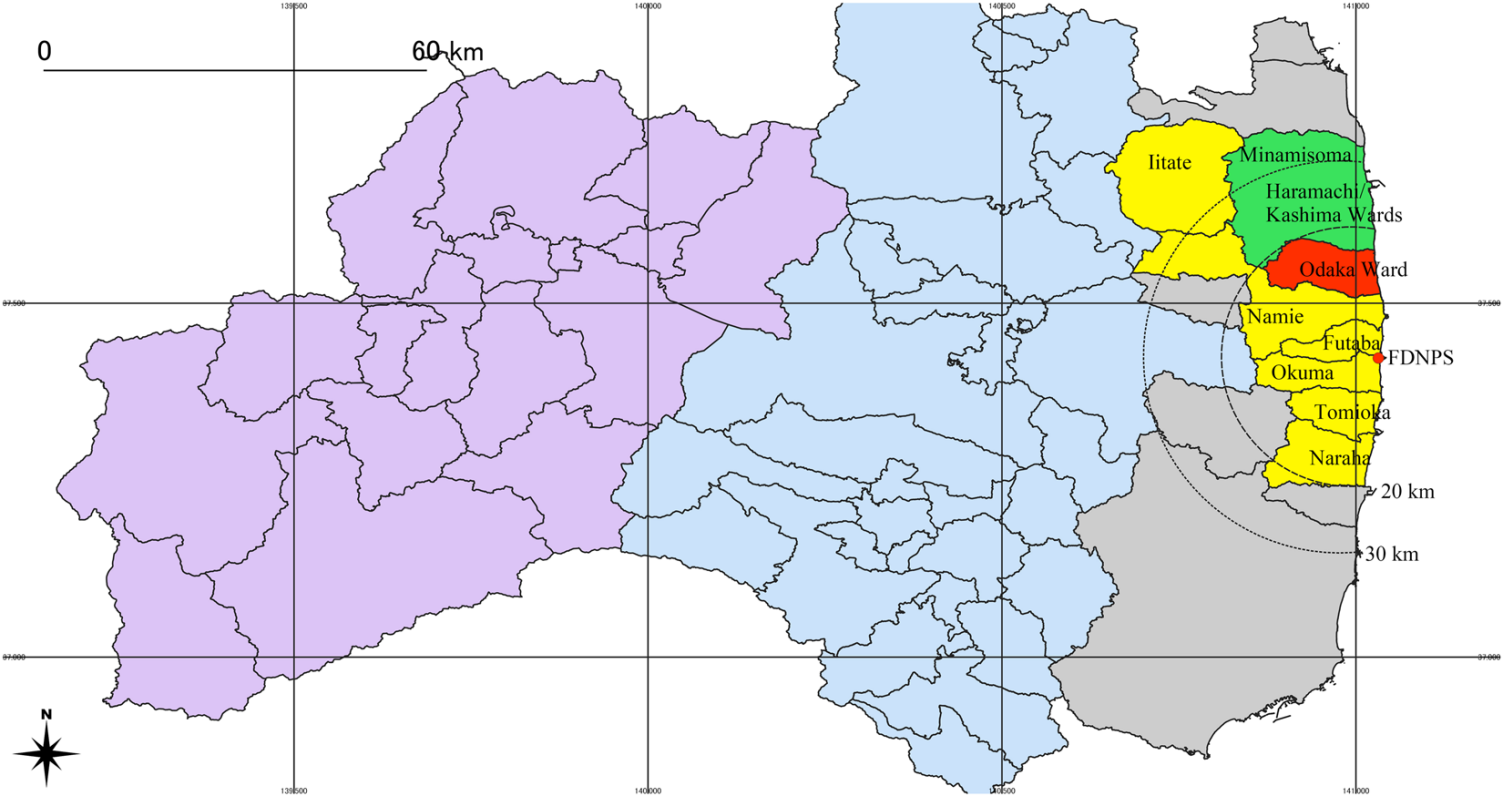
Location of analysed seven municipalities. Location of seven municipalities under evacuation order are shown: Naraha Town, Tomioka Town, Okuma Town, Futaba Town, Namie Tawn and Iitate Village (yellow), and Odaka ward (red) and Haramachi and Kashima wards (green) of Minamisoma City. The remaining areas of Fukushima Prefecture are classified into three areas as below: (1) Soso district including Shinchi Town, Soma City, Katsurao Village, Kawauchi Village, Hirono Town, and Iwaki City (grey), (2) Naka-dori district including Kawamata area, Tamura City, Koriyama City, and Nihonmatsu City (light blue), (3) Aizu district (purple).

**Figure 2:**
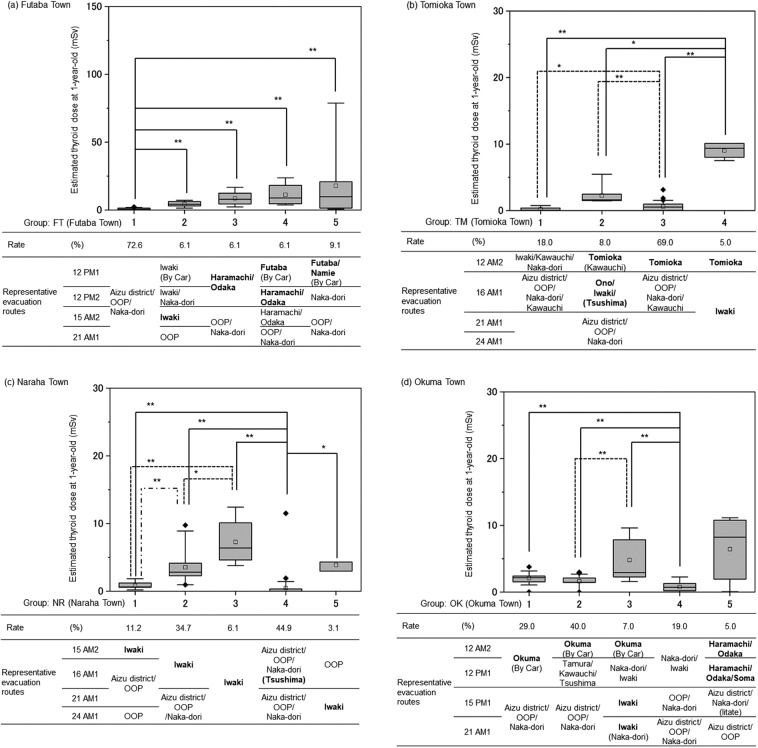
Representative evacuation scenarios for Futaba, Tomioka, Naraha and Okuma towns. Hierarchical clustering analyses by the Ward method were performed for behaviour records of children and youths less than 20 years old to depict representative evacuation scenarios for each municipality with usage rate of the scenario. Then, thyroid doses of 1-year-old children in each evacuation scenario were compared by the Kruskal-Wallis and *post-hoc* Steel-Dwass tests. *p < 0.05, **p < 0.01. The following keys, either doses or places, were used for hierarchical clustering analysis. (**a**) For Futaba town, 12 PM1, 12 PM2, 15 AM2, and 21 AM1 of March 2011. (**b**) For Tomioka town, 12 AM2, 16 AM1, 21 AM1, and 24 AM1 of March 2011. (**c**) For Naraha town, 15 AM2, 16 AM1, 21 AM1, 24 AM1 of March 2011. (**d**) For Okuma town, 12 AM2, 12 PM1, 15 PM1, and 21 AM1 of March 2011. Bold fonts are the key location of suspected exposure from the plumes. OOP: Out of prefecture.

**Figure 3:**
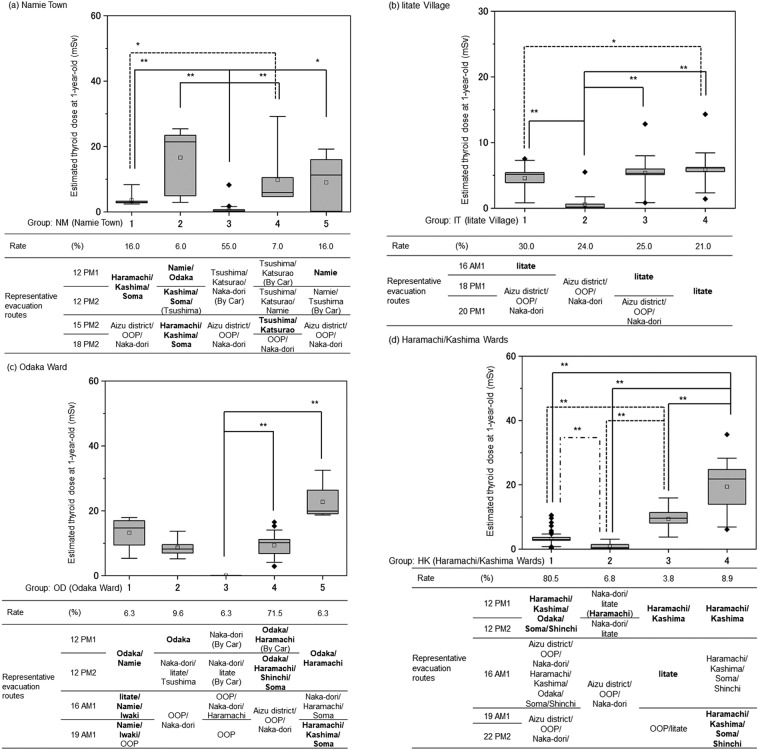
Representative evacuation scenarios for Namie town, Iitate village and Minamisoma city. The following keys, either doses or places, were used for hierarchical clustering analysis. (**a**) For Namie Town, 12 PM1, 12 PM2, 15 PM2, and 18 PM2 of March. (**b**) For Iitate Village, 16 AM1, 18 PM1, and 20 PM1 of March 2011. (**c**) For Odaka ward of Minamisoma City, 12 PM1, 12 PM2, 16 AM1, and 19 AM1 of March 2011. (**d**) For Haramachi/Kashima ward of Minamisoma City, 12 PM1, 12 PM2, 16 AM1, 19 AM1, and 22 PM2. Bold fonts are the key location of suspected exposure from the plumes. OOP: Out of prefecture.

In the winter season, the wind heads primarily to the east over the Pacific Ocean, but it occasionally changes direction. As reported^26^, there were nine radioactive plumes (plume 1–9 or P1–P9) that flew over the coastal area and inland before 22 March, 2011. In addition, four more plumes, P10 on 22 March, P11 on 24 March, P12 on 25 March, and P13 on 30 March, were recorded. Among them, P1 on the afternoon of 12 March, which headed in the northerly direction, affected evacuees and residents in the northern coastal area (Fig. 1), while P2 on the morning of 15 March and P4 on the morning of 16 March, which headed in the southerly direction, affected evacuees and residents in the southern coastal area. P3 on the evening of 15 March headed in the north-westerly direction and encountered the Tsushima area of Namie Town and Iitate Village. Since children voluntarily evacuated rather quickly from a zone 30 km radius around the FDNPS, only a small fraction of children was exposed to the subsequent plumes P5−P13 (Figs. 2 and 3, Supplementary dataset 1). Estimated ^131^I-TEDs for each of the eight municipalities are shown in Table 2, and evacuation scenarios contributing to high or low TEDs in each municipality are shown in Figs. 2 and 3.

**Table 2:** Estimated ^131^I-TEDs (mSv) due to inhalation intake for 1-year old children after correction for lower iodine uptake by thyroid in Japanese and decontamination factor by sheltering.

Municipality	Futaba Town	Tomioka Town	Naraha Town	Okuma Town	Namie Town	Odaka Ward (Minamisoma City)	Haramachi/Kashima Wards (Minamisoma City)	Iitate Village
Mean (95% UI)^a^	3.6 (0.9, 7.5)	1.1 (0.3, 2.3)	2.1 (0.5, 4.4)	2.1 (0.5, 4.3)	4.0 (1.0, 8.4)	9.8 (2.4, 21)	4.7 (1.2, 9.9)	4.0 (1.0, 8.5)
Median (95% UI)^a^	1.3 (0.3, 2.8)	0.5 (0.1, 1.1)	0.9 (0.2, 1.9)	1.7 (0.4, 3.5)	0.8 (0.2, 1.7)	10 (2.5, 21)	3.2 (0.8, 6.6)	5.1 (1.3, 11)
95^th^ percentile (95% UI)^a^	19 (4.7, 39)	7.4 (1.9, 16)	8.9 (2.2, 19)	7.8 (1.9, 16)	20 (5.0, 42)	19 (4.8, 41)	18 (4.5, 38)	8.0 (2.0, 17)

^a^UI: uncertainty interval. UIs were calculated by Monte Carlo simulation as described in Materials and Methods.

As for Futaba Town (FT; Fig. 2a, Table 2), the mean, median, and 95^th^ percentile of ^131^I-TEDs via inhalation were estimated to be 3.6 mSv, 1.3 mSv, and 19 mSv, respectively, and five evacuation scenarios (FT1–5) are depicted. Children of FT3–5 (in total, 21.3%) evacuated to the north (from Futaba Town to Namie Town, Haramachi and Odaka Wards of Minamisoma City) on the afternoon of 12 March so were exposed to P1. FT2 children (6.1%) evacuated to the south to Iwaki City and so were exposed to P2. Some children in FT2 continued to stay in Iwaki City and so were exposed to P4, P9, P11, and P12. FT1 children (72.6%) evacuated to other areas and so were minimally exposed. Median TEDs in FT1–FT5 were 0.4, 4.2, 7.9, 8.8, and 9.7 mSv, respectively. Differences in TEDs among groups FT2–FT5 were not statistically significant.

As to Tomioka Town (TM; Fig. 2b, Table 2), the mean, median, and 95^th^ percentile of ^131^I-TEDs via inhalation were estimated to be 1.1 mSv, 0.5 mSv, and 7.4 mSv, respectively, and four evacuation scenarios (TM1–TM4) are depicted. TM1 and TM3 children (87% of TM) evacuated early and so were minimally exposed to plumes. TM2 children (8.0%) were exposed to either P2 in Iwaki City or P3 in Ono Town or in the Tsushima area of Namie Town. TM4 children (5.0%) remained in Iwaki City and so were exposed to P2, P4, P9, P11, and P12. Median TEDs in TM1–TM4 were 0.1, 1.6, 0.5, and 9.4 mSv, respectively. TEDs in TM4 were significantly higher than in the others.

As for Naraha Town (NR; Fig. 2c, Table 2), the mean, median, and 95^th^ percentile of ^131^I-TEDs via inhalation were estimated as 2.1 mSv, 0.9 mSv, and 8.9 mSv, respectively, and five evacuation scenarios (NR1–NR5) are depicted. The main representative evacuation pattern in our study was NR4 (44.9% of NR) in which evacuees moved to another area (Aizu district, OOP, and Naka-dori) before 15 March. But, in NR4 one subject was exposed minimally to plumes or exceptionally exposed to P3 in the Tsushima area of Namie Town. Children in other groups were exposed once or repeatedly to P2, P4, P9, P11, and P12 at Iwaki City. Median TEDs in NR1–NR5 were 0.6, 2.8, 6.4, <0.1, and 4.3 mSv, respectively. NR3 children (6.1% of NR) showed higher TEDs than others.

As for Okuma Town (OK; Fig. 2d, Table 2), the mean, median, and 95^th^ percentile of ^131^I-TEDs via inhalation were estimated to be 2.1 mSv, 1.7 mSv, and 7.8 mSv, respectively, and five evacuation scenarios (OK1–OK5) are shown. Children in OK1, OK2, and OK4 (88.0% of OK) had minimal exposure to the plumes, but OK3 children (7.0%) or OK5 children (5.0%) were exposed once or multiple times to P2, P4, P9, P11, and P12 at Iwaki City or to P1, P5, P6, P8, P10, and P13 at Minamisoma and Soma Cities, respectively. Median TEDs in OK1–5 were 2.2, 1.5, 2.9, 0.7, and 8.2 mSv, respectively.

Regarding Namie Town (NM; Fig. 3a, Table 2), the mean, median, and 95^th^ percentile of ^131^I-TEDs via inhalation were estimated to be 4.0 mSv, 0.8 mSv, and 20 mSv, respectively, and five evacuation scenarios (NM1–NM5) are depicted. NM1 children (16.0% of NM) evacuated to Minamisoma and Soma Cities, and were exposed to P1 in the evening of 12 March. NM2 and NM5 (22.0%) were exposed to P1 on 12 March either in Namie Town or in Odaka Ward, and tended to show relatively high TEDs. It was noted that children in the NM3 group (55.0%) evacuated to distant areas via the Tsushima area or Katsurao Village before P1 reached Namie Town and were only minimally exposed to the plumes. NM4 children (7.0%) evacuated to Tsushima and were exposed to P3 on 15 March. Median TEDs in NM1–NM5 were 2.9, 21, 0.3, 5.9, and 11 mSv, respectively.

As for Iitate Village (IT; Fig. 3b, Table 2), the mean, median, and 95^th^ percentile of ^131^I-TEDs via inhalation were estimated to be 4.0 mSv, 5.1 mSv, and 8.0 mSv, respectively, and four evacuation scenarios (IT1–IT4) are shown. Children in the IT2 group (24.0% of IT) were evacuated early and so were not exposed to P3 at night on 15 March and in the early morning on 16 March, whereas other groups were exposed to this plume. Some areas in Iitate Village were affected by P5 and P8, but the doses delivered were generally small. Median TEDs via inhalation in IT1–IT4 were 5.2, 0.2, 5.3, and 6.1 mSv, respectively. TEDs of the IT4 group were significantly higher than those of the IT1 (30.0%) and IT2 (24.0%) groups, but did not differ significantly from those of the IT3 group (25.0%). Because tap water contamination was significant in Iitate Village, TEDs via ingestion differed among the groups, as discussed later.

Regarding Odaka Ward of Minamisoma City (OD; Fig. 3c, Table 2), the mean, median, and 95^th^ percentile of ^131^I-TEDs via inhalation were estimated as 9.8 mSv, 10 mSv, and 19 mSv, respectively, and five evacuation scenarios (OD1–OD5) are depicted. Children in Odaka Ward showed higher mean and median TEDs than children in other municipalities (Table 2). This was because 93.4% of children—with the exception of OD3 in Odaka Ward—were exposed to P1 at either Namie Town or Odaka Ward before evacuation or after evacuation at either Haramachi or Kashima Wards or Soma City at night on 12 March. Children in the OD1 (6.3%) and OD5 (6.3%) groups were exposed to P1 and additionally to P3 at Iitate Village or in the Tsushima area of Namie Town, or to P2 and P4 in Iwaki City (OD1), and to P5 and P6 in Minamisoma or Soma Cities (OW5), respectively. Median TEDs via inhalation in OD1–OD5 were 15, 8.2, <0.1, 10, and 20 mSv, respectively. The TEDs of evacuation pattern OD3 (6.3%) were significantly lower than those of OD4 (71.5%) and OD5 (6.3%).

As for Haramachi and Kashima Wards of Minamisoma City (HK; Fig. 3d, Table 2), the mean, median, and 95^th^ percentile of ^131^I-TEDs via inhalation were estimated to be 4.7 mSv, 3.2 mSv, and 18 mSv, respectively, and four evacuation scenarios (HK1–HK4) are depicted. Children in HK2 (6.8% of HK) were only minimally exposed to plumes because of early evacuation. HK1 children (80.5%) were exposed to P1 but were evacuated before 18 March when P5 reached the northern coastal area. Children in HK4 (8.9%) were exposed to P1 and additional plumes either on 18, 19, 20, 22, or 30 March 2011. Median TEDs via inhalation in HK1–HK4 were 3.0, 0.4, 9.6, and 22 mSv, respectively.

### TEDs via inhalation of ^131^I, ^132^Te/^132^I and ^133^I

Total TEDs via inhalation of ^131^I, ^132^Te/^132^I, and ^133^I were estimated by utilizing the relative nuclide compositions of P1 and P2 as estimated in the previous study^15^ (Table 3). Total TEDs increased, especially in Futaba and Namie Towns and in Odaka Ward, since a sizable fraction of children in these municipalities were exposed to P1. However, the highest 95^th^ percentiles of TEDs were 30 mSv in Futaba and Namie Towns, and their upper bounds of 95% U.I. (uncertainty interval) were 63 and 62 mSv, respectively.

**Table 3:** Estimated TEDs (mSv) of 1-year-old children via inhalation of ^131^I, ^132^Te/^132^I, and ^133^I^a^.

	Futaba Town	Tomioka Town	Naraha Town	Okuma Town	Namie Town	Odaka ward (Minamisoma City)	Haramachi/Kashima wards (Minamisoma City)	Iitate Village
Mean	5.3	1.2	2.3	2.9	5.7	15	6.3	4.5
(95%UI)^b^	(1.3, 11)	(0.3, 2.5)	(0.6, 4.7)	(0.7, 6.0)	(1.4, 12)	(3.7, 31)	(1.6, 13)	(1.1, 9.3)
Median	1.5	0.6	1.0	2.4	0.9	16	4.8	5.6
(95%UI)^b^	(0.4, 3.1)	(0.1, 1.2)	(0.3, 2.1)	(0.6, 5.1)	(0.2, 2.0)	(4.0, 33)	(1.2, 10)	(1.4, 12)
95^th^ pepcentile	30	7.5	9.7	9.1	30	25	19	9.1
(95%UI)^b^	(7.5, 63)	(1.9, 16)	(2.4, 20)	(2.3, 19)	(7.4, 62)	(6.3, 53)	(4.9, 41)	(2.3, 19)

^a^Method of TEDs via inhalation of ^131^I, ^132^Te/^132^I, and ^133^I is described in Materials and Methods.

^b^UI: uncertainty interval.

## Discussion

In the present study, TEDs were estimated on the basis of randomly sampled individual behaviour questionnaire survey sheets and the revised version of the spatiotemporal radionuclides concentration database, WSPEEDI_2019DB, which was improved by referring to hourly measured ^137^Cs concentrations in air at multiple SPM stations^26^. In addition, two factors that influence TEDs were incorporated into the present dose estimation: a decontamination factor for sheltering and a correction factor for dose coefficient to reflect the lower thyroid iodine uptake rate in Japanese^18,24^. By these procedures, we estimated ^131^I-TEDs as realistically as possible. To validate our estimates, it is essential to compare simulated doses with those based on direct thyroid measurements. Kim *et al*. conducted direct thyroid measurements in 1,080 children from Iwaki City, Kawamata Town, and Iitate Village in March 2011 with an NaI(Tl) scintillation survey meter^11^. Among those children, 31 were from Minamisoma City. Tokonami *et al*. conducted direct thyroid measurements using an NaI(Tl) spectrometer for 64 evacuees or residents from Odaka Ward, Minamisoma City, and the Tsushima area of Namie Town in April 2011^10^. In the original reports, these authors supposed that acute exposure occurred on 15 March, but in the present analyses we re-calculated the TEDs of evacuees from Odaka Ward and Minamisoma City under the assumption that acute exposure occurred from P1 on 12 March (Table 4). In the case of evacuees from Iitate Village, inhalation dose and ingestion dose from tap water were calculated on the basis of 100 whereabouts questionnaires (Table 4). It should be noted that 0 mSv (25^th^ percentile figures in Table 4) does not necessarily imply no ^131^I activities in the thyroid, but rather reflects the difficulty in detecting a small amount of thyroid activities under the background counts of 0.12 μSv/h in Iitate Village^11^. As demonstrated in Table 4, our TED estimates via inhalation for one-year-old children from Minamisoma City and for adults from Odaka Ward are generally consistent with values reported by Kim *et al*.^11^ and Tokonami *et al*.^10^, respectively. If a few mSv of additional radionuclide ingestion via tap water is considered^20,21^, our central estimates for evacuees from Minamisoma City might be a little bit larger than the measured values, but they are within the 95% U.I. As for Iitate Village, our estimates are very close to values obtained by direct measurements reported by Kim *et al*.^11^. As to subjects exposed to plume(s) in Iwaki city, Morita *et al*. reported TEDs for 16 adults based on individual ^131^I activities detected by a whole-body counter, who evacuated from Hirono Town on 11 March and left Iwaki City on either 15 or 16 March^27^. Their TEDs ranged from 0.6 to 5.8 mSv (mean = 1.5 mSv). As the evacuation scenario of these 16 subjects was like NR2 evacuation scenario in which 34 children evacuated from Naraha Town to Iwaki City on 12 March and left Iwaki City on 16 March or a few days later (Fig. 2c). Our estimated TEDs for 1-year-old children following NR2 scenario ranged from 0.9 to 9.8 mSv (mean: 3.52 mSv (95%UI: 0.9, 7.4)). As adult TED via inhalation is about the half of the TED of 1-year-old child, our estimated TEDs for children following NR2 scenario are consistent with those estimates by Morita^27^. These results demonstrate that the present dose estimates would not differ from actual doses by a factor of two if 95% U.I. is considered.

**Table 4:** Comparison between estimated ^131^I-TEDs by simulation and by direct thyroid measurements.

Methodology	Simulation			Direct thyroid measurement							
Reference	^131^I-TEDs (mSv) in this study			^131^I-TEDs estimates (mSv)^11^				^131^I-TEDs estimates (mSv)^10^			
	25^th^ percentile (95%U.I.)	Median (95%U.I.)	75^th^ percentile (95%U.I.)	(N)	25^th^ percentile	median	75^th^ percentile	(N)	25^th^-percentile	median	75^th^-percentile
Minamisoma city, 1-year-old, inhalation dose	2.7 (0.7, 5.7)	3.5 (0.9, 7.4)	7.4 (1.7, 15)	(31)	0	3.9*	11*	N.E.^#^			
Odaka ward, 1-year-old, inhalation dose	6.6 (1.6, 14)	10 (2.5, 21)	12 (3.0, 25)								
Odaka ward, adult, inhalation dose	3.3 (0.8, 7)	5 (1.3, 11)	6 (1.5, 13)	N.E.				(32)	1.6^$^	4^$^	6^$^
Iitate village, 5-years-old, inhalation and ingestion dose	1.5 (0.4, 2.9)	7 (1.9, 15)	14 (4.7, 26)	(99)	0	7.3	14.7	N.E.			
Iitate village, 10-years-old, inhalation and ingestion dose	1.2 (0.4, 2.5)	6.1 (1.6, 12)	12 (3.7, 22)	(114)	0	3.7	7.5				

*Exposure day was set as 12 March, instead of 15 March, 2011 as in the manuscript by Kim *et al*.^11^, and thus figures were multiplied by 1.4 as in Kim *et al*.^11^, as described in the manuscript. The age distribution of 31 subjects was not known.

^$^Exposure day was set as 12 March, instead of 15 March, 2011 as in the manuscript by Tokonami *et al*.^10^ and individual data were recalculated. One family (5 members) was omitted from the current reanalysis because they remained at Namie town until the end of March, 2011, and were surely exposed to plumes more than once.

^#^Not estimated.

In the UNSCEAR 2013 report, only one or two representative evacuation scenarios per municipality were utilized to estimate thyroid absorbed doses. Thus, the dose distribution among evacuees from the same municipality could not be illustrated. So far, an epidemiological study of thyroid cancer in Fukushima has been conducted as an ecological study that compared thyroid cancer incidence in four districts of different exposure levels or municipalities by using the doses of the UNSCEAR 2013 report as surrogate ‘personal’ doses^28^. Dose estimation based on personal whereabouts information will be useful for conducting case-control studies that would not be subject to confounding and biases inherent in ecological studies. In the present study, we have established a methodology for personal dose estimation and depicted several evacuation scenarios with different dose levels for each municipality. These methodology and data will be valuable for future thyroid studies in Fukushima.

The present study has several limitations. First, ATDM simulation intrinsically contains uncertainties about source terms and meteorological data used in the simulation although our ATDM reproduced most measurements successfully in comparison with the previous studie^13^. We tried to reduce uncertainty in the spatiotemporal distributions of radionuclides by averaging ^131^I concentrations during 6-hour periods, but it induced another uncertainty. For example, even if evacuees left before the plume arrival in the afternoon of 12 March, they were assumed to have inhaled air containing an averaged concentration of ^131^I from 12:00 to 18:00. Second, we could not estimate TEDs on or after 26 March 2011, because the detailed version of the behaviour questionnaire encompassed behaviour from 11 to 25 March^16^. From 26 March, evacuees and residents in Iwaki City might have been exposed to P12. Likewise, those in the Haramachi and Kashima Wards of Minamisoma City and in Soma City might have been exposed to P13. However, ^131^I-concentrations in these municipalities were generally low judging from the ATDM simulation^13^ (Supplementary dataset 2). Third, whereabouts questionnaire data might be inaccurate as evacuees reported the questionnaire several months later. In addition, most people did not report exact road numbers taken or resting places during evacuation, which would have been informative for dose estimation. Fourth, we could not assess individual housing conditions—such as windows or sliding doors being open or closed—when the plumes came. However, March 2011 was still in the winter season in Japan and a sheltering order was announced in the 30 Km zone from FDNPS. Thus, it is reasonable to utilize the *DF*_*shelter*_ experimentally deduced from houses under natural ventilation conditions^24^. It is noted that the decontamination factor ranges from 0.1 to 0.95, and old housing offers less protection from the plumes. Fifth, we could not assess individual dietary habits or intake of stable iodine tablets. If iodine-rich food or stable iodine tablets were consumed within 2 days before plume exposure, accumulation of radio-iodine in the thyroid gland would be suppressed. However, such individual information was not available or was obtained only in exceptional cases. Instead of assessing individual dietary habits, we adopted the distribution of iodine uptake rate reported by Kudo, who experimentally assessed the rate among normal subjects without iodine restriction^18^. Sixth, *SF*, a correction factor for short-lived radionuclides, might vary from place to place on 12 March, as P1 was a mixture of two plumes released by venting at 14:30 and by a hydrogen explosion at 15:36, and venting might deplete low volatile ^132^Te in the plume. Finally, we assumed that randomly sampled questionnaire sheets represent the diversity of evacuation scenarios in all age groups, but it is uncertain whether age-specific scenarios are more appropriate or not. In spite of these limitations, estimated TEDs in the present study are consistent with doses based on direct thyroid measurements.

## Conclusion

After uncertainties in dose estimation were reduced—by improving the ATDM simulation, by re-evaluation of evacuation scenarios, by introducing a decontamination factor for sheltering in Japanese houses, and by introducing a correction factor for dose coefficient to reflect lower iodine uptake ratio by thyroid in Japanese—estimated TEDs via inhalation after the FDNPS accident were very close to doses estimated from direct thyroid measurements. The median and 95^th^ percentile of TEDs in 1-year-old children ranged from 0.6 to 16 mSv, and 7.5 to 30 mSv, respectively. Our estimates are much smaller than those in the UNSCEAR 2013 report.

## Supplementary information

Supplementary Figures, Tables and Memorandum for Datasets.

Dataset 1.

Dataset 2.

## Data Availability

A source term for the ATDM simulation and WSPEEDI_2019DB written according to a NetCDF protocol are available from the WEB site of the *J. Env. Radioactivity*^13^. Representative evacuation routes with latitudes and longitudes for municipalities are available as Supplementary dataset 1. An Excel file is available as Supplementary dataset 2 that depicts selected radionuclide concentrations (Bq/m^3^) at 1 m height at 152 landmarks from WSPEEDI_2019DB. The Radiation Medical Science Centre of Fukushima Medical University authorised us to analyse the current data of the Basic Survey from the FHMS in this study. The centre currently restricts usage of FHMS data to members or observers of special committees of the FHMS. T.O., T.I., A. H., and G.S. are observers in such special committees. The authors are not allowed to provide the whereabouts questionnaire data to a third party. However, the Committee on the Fukushima Health Management Survey promulgated in July 2019 general guidelines for providing FHMS data for research purposes. Detailed regulations are under consideration, and FHMS data without personal information will be available upon request after Committee review of a request in the near future. Please contact Citizens Healthcare Survey Division, Social Health and Welfare Department, Fukushima Prefectural Government. Address: 2–16 Sugitsuma-cho, Fukushima City, Fukushima Prefecture, Japan, zip code 960–8670.
