# Author Correction: Reconstruction of residents’ thyroid equivalent doses from internal radionuclides after the Fukushima Daiichi nuclear power station accident

**DOI:** 10.1038/s41598-020-62931-x

**Published:** 2020-04-07

**Authors:** Takashi Ohba, Tetsuo Ishikawa, Haruyasu Nagai, Shinji Tokonami, Arifumi Hasegawa, Gen Suzuki

**Affiliations:** 1 Department of Radiation Health Management, School of Medicine, Fukushima Medical University, Fukushima-city, Fukushima 9601295 Japan; 2 Radiation Medical Science Centre for the Fukushima Health Management Survey, Fukushima Medical University, Fukushima-city, Fukushima 9601295 Japan; 3 Department of Radiation Physics and Chemistry, School of Medicine, Fukushima Medical University, Fukushima-city, Fukushima 9601295 Japan; 4 Environment and Radiation Science Division, Japan Atomic Energy Agency, Tokai-village, Ibaraki 3191195 Japan; 5 Department of Radiation Physics, Institute of Radiation Emergency Medicine, Hirosaki University, Hirosaki-city, Aomori 0368564 Japan; 6 Department of Radiation Disaster Medicine, School of Medicine, Fukushima Medical University, Fukushima-city, Fukushima 9601295 Japan; 7 International University of Health and Welfare Clinic, Ohtawara-city, Tochigi 3248501 Japan

Correction to: *Scientific Reports* 10.1038/s41598-020-60453-0, published online 27 February 2020

This Article contains errors in the Methods section under subheading ‘Estimation of TED via inhalation’ where,

“*FC* is a correction factor for the dose coefficient because iodine uptake rate is 18.5% (SD 6.0%), lower than the 30% in the ICRP thyroid model, while thyroid volume in Japanese does not differ from that of ICRP reference man^18^. *DF*_*shelter*_ is a decontamination factor to reflect sheltering. In the present study we set FC to 0.62 (=18.5/30) and *DF*_*shelter*_ to 0.5, and the combined uncertainty interval of these two factors is simulated as described below.”

should read:

“*FC* is a correction factor for the dose coefficient because iodine uptake rate is 18.6% (SD 6.0%), lower than the 30% in the ICRP thyroid model, while thyroid volume in Japanese does not differ from that of ICRP reference man^18^. *DF*_*shelter*_ is a decontamination factor to reflect sheltering. In the present study we set FC to 0.62 (=18.6/30) and *DF*_*shelter*_ to 0.5, and the combined uncertainty interval of these two factors is simulated as described below.”

The Article also contains an error in equation (3) in the Methods section under the subheading ‘Estimation of TED via ingestion in Iitate village’ where,

$${{\rm{E}}}_{{\rm{Thyroid}}({\rm{ingestion}})}=\mathop{\sum }\limits_{{\rm{j}}}^{14}{{\rm{V}}}_{{\rm{tap}}}\times {{\rm{C}}}_{{\rm{tap}}}\times {{\rm{e}}}_{{\rm{ing}}/{\rm{thy}}}\times {\rm{FC}}\times {\rm{Sf}}\times \frac{{{\rm{X}}}_{{\rm{j}}}}{3}$$

should read:

$${{\rm{E}}}_{{\rm{Thyroid}}({\rm{ingestion}})}=\mathop{\sum }\limits_{{\rm{j}}}^{14}{{\rm{V}}}_{{\rm{tap}}}\times {{\rm{C}}}_{{\rm{tap}}.{\rm{j}}}\times {{\rm{e}}}_{{\rm{ing}}/{\rm{thy}}}\times {\rm{FC}}\times {\rm{Sf}}\times \frac{{{\rm{X}}}_{{\rm{j}}}}{3}$$

In addition, there is a typographical error in the Methods section under subheading ‘Uncertainty of correction factors’ where,

“Likewise, the combined uncertainty of *FC* and *Sf* in formula (2) was simulated by setting *FC* as having a normal distribution with (18.6 +/−6.0)/30% and *Sf* as having a binomial distribution with denominator 100 and expected proportion 0.3.”

should read:

“Likewise, the combined uncertainty of *FC* and *Sf* in formula (3) was simulated by setting *FC* as having a normal distribution with (18.6 +/−6.0)/30% and *Sf* as having a binomial distribution with denominator 100 and expected proportion 0.3.”

Finally, this Article contains errors in References 18 and 28 which are incorrectly given as:

18. Kudo, T. et al. Determination of the kinetic parameters for 123I uptake by thyroid, and thyroid weights and volumes, In present-day healthy Japanese volunteers*. Health phys*. In press (2019).

28. Fukushima Revitalization Station (Fukushima Prefecture Government). Relationship between estimated thyroid absorbed doses based on UNSCEAR 2013 report and the rate of detection of malignant or suspected malignancy by each municipality (No. 1-2), on 3 June 2019, http://www.pref.fukushima.lg.jp/site/portal/kenkocyosa-kentoiinkai-b13.html (2019).

The correct references are listed below as References 1 and 2:
