# Supplementary material for: Reconstruction of residents’ thyroid equivalent doses from internal radionuclides after the Fukushima Daiichi nuclear power station accident
Source: Sci Rep. 2020 Feb 27;10:3639. doi: 10.1038/s41598-020-60453-0 (PMC7046762; doi:10.1038/s41598-020-60453-0)
Supplement: Supplementary file 1 — Supplementary Figures, Tables and Memorandum for Datasets. [file 41598_2020_60453_MOESM1_ESM.pdf]

Electronic supplementary material

Title: Reconstruction of residents' thyroid equivalent doses from internal radionuclides after the Fukushima Daiichi nuclear power station accident

Author list and affiliations;

Takashi Ohba[1], Tetsuo Ishikawa[2,3], Haruyasu Nagai[4], Shinji Tokonami[5], Arifumi Hasegawa[6], and Gen Suzuki[7]*

[1]Department of Radiation Health Management, School of Medicine, Fukushima Medical University, Fukushima-city, Fukushima, 9601295, Japan

[2]Radiation Medical Science Centre for the Fukushima Health Management Survey, Fukushima Medical University, Fukushima-city, Fukushima, 9601295, Japan

[3]Department of Radiation Physics and Chemistry, School of Medicine, Fukushima Medical University, Fukushima-city, Fukushima, 9601295, Japan

[4]Environment and Radiation Science Division, Japan Atomic Energy Agency, Tokai-village, Ibaraki, 3191195, Japan

[5]Department of Radiation Physics, Institute of Radiation Emergency Medicine, Hirosaki University, Hirosaki-city, Aomori 0368564, Japan,

[6]Department of Radiation Disaster Medicine, School of Medicine, Fukushima Medical University, Fukushima-city, Fukushima, 9601295, Japan

[7]International University of Health and Welfare Clinic, Ohtawara-city, Tochigi, 3248501, Japan

*Correspondence and requests for materials should be addressed to G.S. (email: gensuzki@iuhw.ac.jp)

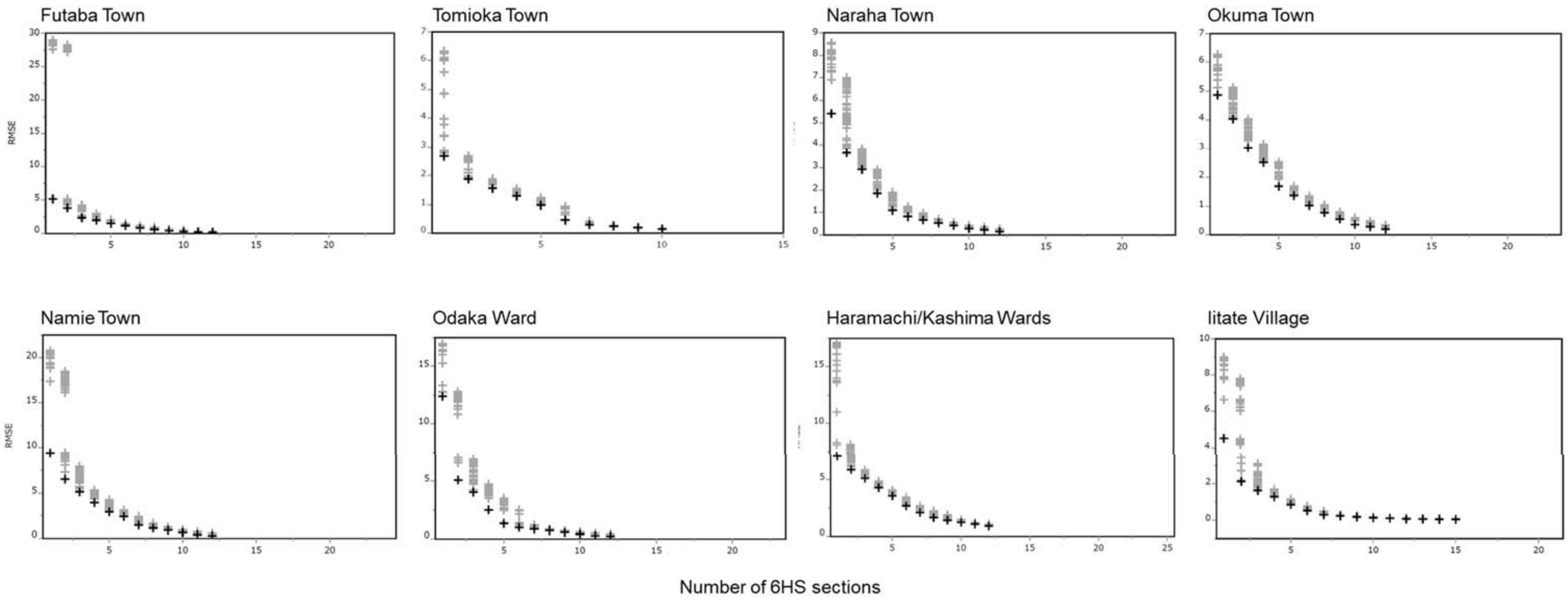

Supplementary Figure 1. The tendency of root mean squared error (RMSE) with number of 6HS sections by step-wise regression analyses.

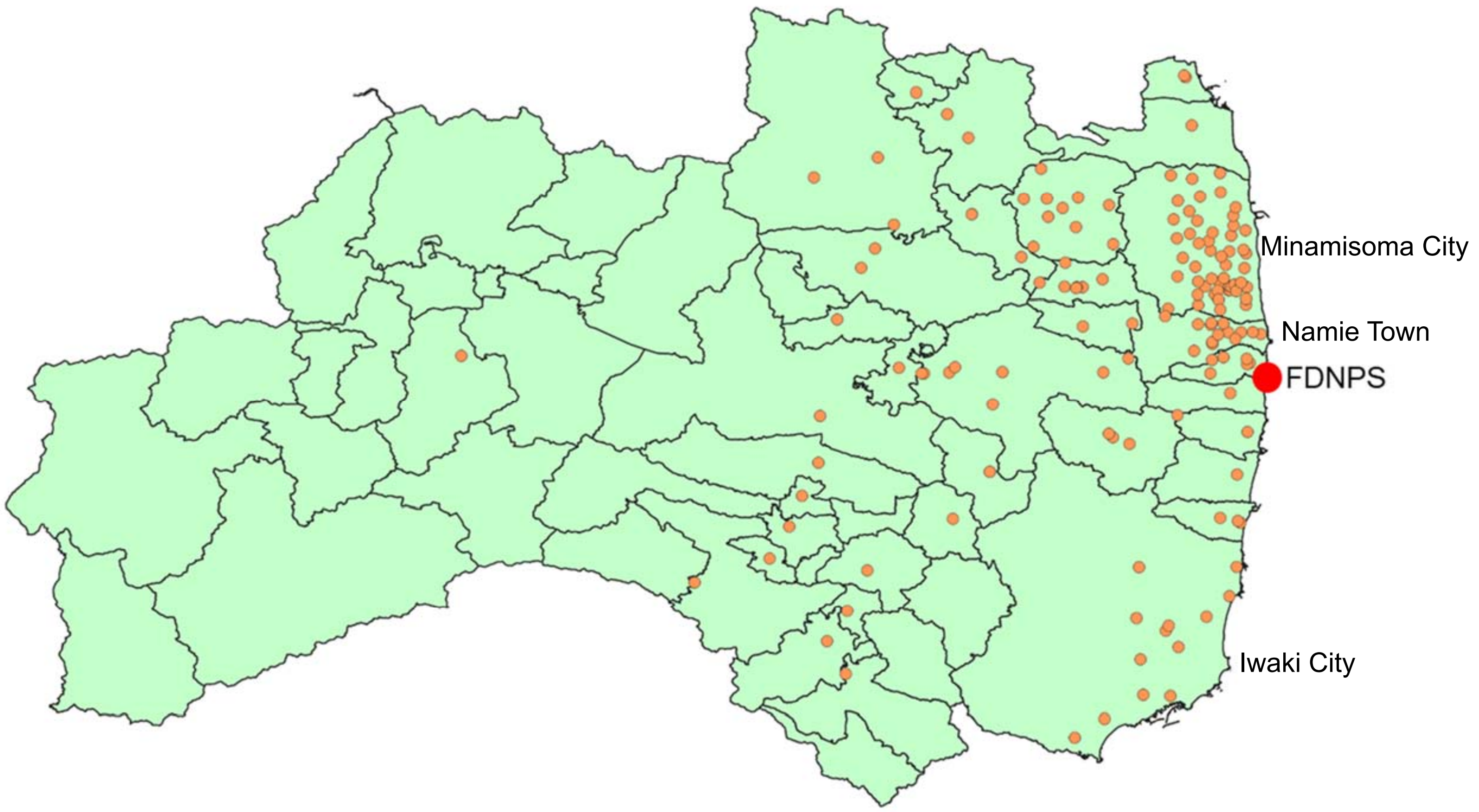

Supplementary Figure 2. Locations of 152 landmarks (orange circles) in Fukushima Prefecture that were used to estimate thyroid doses.

Supplementary Table 1. List of 18 evacuation scenarios reported in the 2013 UNSCEAR report [2].

No	Location at 11th March 2011	Main evacuation behaviour in March 2011
#1	Tomioka Town	12 March: Kawauchi Village, 16 March: Koriyama City
#2	Okuma Town	12 March: Tamura City
#3	Futaba Town	12 March: Kawamata Town at 08:00, 19 March: Out of Fukushima Prefecture
#4	Futaba Town	12 March: Kawamata Town at 21:00, 19 March: Out of Fukushima Prefecture
#5	Naraha Town	12 March: Iwaki City, 31 March: Tamura City
#6	Naraha Town	12 March: Iwaki City, 16 March: Aizu-area
#7	Namie Town	12 March: Namie Town (Tsushima-area), 16 March: Nihonmatsu City
#8	Tamura City	31 March: Koriyama City
#9	Minamisoma City (Odaka Ward)	15 March: Date City, 31 March: Fukushima City
#10	Hirono Town	12 March: Ono Town
#11	Kawauchi Village	16 March: Koriyama City
#12	Katsurao Village	14 March: Fukushima City
#13	Namie Town (Tsushima-area)	23 March: Nihonmatsu City
#14	Katsurao Village	21 March: Fukushima City
#15	Iitate Village	Fukushima City on May 2011
#16	Iitate Village	Fukushima City on June 2011
#17	Minamisoma City (Haramachi/Kashima Wards)	Minamisoma City on May 2011
#18	Kawamata Town (Yamakiya-area)	Kawamata Town on June 2011

Supplementary Table 2. Conversion factor for ^{131}I to calculate estimated thyroid equivalent dose for 1-year-olds, 5-year-olds, 10-year-olds, 15-year-olds, and adults. A) Inhalation with ventilation rate based on ICRP publication 71 [17]; B) Ingestion based on ICRP publication 67 [23].

**A**	**Conversion factor**			**Time after intake**	**Ventilation rate**
	**Methyl form (^{131}I)**	**Elemental vapor form (^{131}I)**	**Particle aerosol (^{131}I), AMAD=1.0 μm, Type F**		
**Unit**	**Sv/Bq**			**Years**	**m^3/day**
1-year-old	2.5×10^{-6}	3.2×10^{-6}	1.4×10^{-6}	69	5.16
5-year-old	1.5×10^{-6}	1.9×10^{-6}	7.3×10^{-7}	65	8.72
10-year-old	7.4×10^{-7}	9.5×10^{-7}	3.7×10^{-7}	60	15.3
15-year-old	4.8×10^{-7}	6.2×10^{-7}	2.2×10^{-7}	55	20.1 (male)
Adult	3.1×10^{-7}	3.9×10^{-7}	1.5×10^{-7}	50	22.2 (male)

**B**	**Conversion factor**	**Time after intake**
	**^{131}I、f1=1.0**	
**Unit**	**Sv/Bq**	**Years**
1-year-old	3.6×10^{-6}	69
5-year-old	2.1×10^{-6}	65
10-year-old	1.0×10^{-6}	60
15-year-old	6.8×10^{-7}	55
Adult	4.3×10^{-7}	50

Supplementary Table 3. Construction-year-averaged $DF_{shelter}$ scores at different wind speeds for an elapsed time of 6 hours in Japanese houses.

**Construction year of house**	**Fraction of houses in Fukushima Prefecture in 2008 [25]**	***$DF_{shelter}$* score for 6 hours elapsed time under different wind speeds (m/s) in houses constructed under different building codes adopted from Hirouchi's report [24]**		
		**1.0**	**4.0**	**2.5**
pre-1980	0.40	0.55	0.95	0.75
1980-1992	0.20	0.15	0.78	0.47
post-1992	0.40	0.10	0.43	0.26
Construction-year-averaged $DF_{shelter}$ score		0.29	0.70	0.50

Construction-year-averaged $DF_{shelter}$ scores are the weighted means of $DF_{shelter}$ scores by sheltering in houses constructed under either pre-1980, 1980, or 1992 building codes.

Memorandum for Supplementary datasets

Dataset 1:
These data demonstrate 37 “typical” evacuation patterns of representative evacuation scenarios described in the manuscript. Latitudes and longitudes indicate places he or she visited during evacuation at indicated 6-hour-segment (6HS). FT; Futaba town, TM; Tomioka town, NR; Naraha town, OK; Okuma town, NM; Namie town, IT; Iitate village, OD; Odaka ward of Minamisoma city, HK; Haramachi and Kashima wards of Minamisoma city.

Dataset 2:
Data describe radionuclides concentration (Bq/m3) at 1 m height at 152 landmarks.
These data are depicted from WSPEEDI_2019DB published by H. Terada et al. (Refinement of source team and atmospheric dispersion simulations of radionuclides during the Fukushima Daiichi Nuclear Power Station accident. *J. Environ. Radioact.* Doi: 10.1016/j.jenvrad.2019.106104), which demonstrate the temporal pattern of ^{137}Cs, ^{134}Cs, ^{131}I_particle, ^{131}I_I_2 gas and ^{131}I$_{_methyl}$ and ^{131}I_Total concentrations (Bq/m^3) at 1m height from land surface at 152 landmarks. “Wspeedi2019L” and “Wspeedi2019R” represent data obtained by local scale simulation (187km x 187km, horizontal resolution of 1km) and regional simulation (381km x 561km, horizontal resolution of 3km), respectively. “Lon” and “lat” in the table represent the longitude and latitude of a landmark, respectively. “Lon_sim” and “Lat_sim” represent the longitude and latitude of the nearest simulated point by WSPEEDI, respectively, and “dis” shows the distance from the landmark to the simulated point.
